# A hierarchical dataset of vegetative and reproductive growth in apple tree organs under conventional and non-limited carbon resources

**DOI:** 10.1016/j.dib.2023.109011

**Published:** 2023-02-27

**Authors:** F. Reyes, M. Tagliavini, D. Gianelle

**Affiliations:** a University of Studies of Modena and Reggio Emilia, Life Science Department, via Amendola 2, 42122, Reggio Emilia, Italy; b Free University of Bozen-Bolzano, Faculty of Science and Technology, Piazza Università, 5, 39100, Bozen-Bolzano, Italy; c Forest Ecology Unit, Research and Innovation Centre, Fondazione Edmund Mach, Via E. Mach 1, San Michele All'adige, 38010 Trento, Italy

**Keywords:** Shoot, Fruit, Trunk, Hierarchical sampling, Growth parameters, Maximum potential growth, Carbon availability, Modeling

## Abstract

A monitoring of apple fruit, shoot and trunk growth was performed on 15 trees, equally split according to three treatments, which determined heavily contrasting carbon assimilate availability: unmanipulated trees (FRU), thinned trees (THI) and defruited trees (DEF).

Several variables describe the vegetative growth on FRU and DEF trees (shoot length, base diameter, number of fruits on shoot, and height, diameter, pruning intensity and number of fruits of the branch carrying the shoot; trunk circumference), as well as the fruit growth on FRU and THI trees (3 fruit diameters). Additional measurements from ancillary shoots (apical diameter, number of leaves, leaf dry weight, stem dry weight, fresh mass, volume) and fruits (3 diameters, dry weight) from trees undergoing the same treatments, provide a more complete (destructive) characterization of organs growth, thanks to several measurements performed across the growing season. Organs are provided with categorical variables indicating the treatment, tree, canopy height, orientation (for both shoots and fruit), as well as branch and shoot identifiers, so that hierarchical modeling of the dataset can be performed. The dataset is completed with dates and day of the year of the measurements and the accumulated growing degree days from full bloom. Data can be used to calculate apple tree absolute and relative growth rates, maximum potential growth rates, as well as shoot growth responses to thinning and pruning. The dataset can also be used to calibrate allometric relationships, estimate structural apple tree growth parameters and their variability.

**Specifications Table**

**Table:** 

Subject	Agronomy and Crop Science

Specific subject area	Temperate fruit tree crops
Type of data	Table
How the data were acquired	Tree organs morphology monitoring via manual instruments: caliper, tape measure, precision scale after oven drying, submersion in graduated cylinder.
Data format	Raw
Description of data collection	Data were collected from trees with greatly different fruit load: notably from trees completely defruited or heavily thinned three weeks after bloom, and from trees in normal commercial field conditions.
Data source location	Location: an organic commercial orchard City: Caldaro, Bolzano/Bozen province, Trentino Alto Adige region Country: Italy Latitude and longitude collected samples/data: 46° 21’ N, 11° 16’ E, Altitude 240 m Period: May-November 2014
Data accessibility	Repository name: Mendeley Data Data identification number: 10.17632/852r5dnzd5.1 Direct URL to data: https://data.mendeley.com/datasets/852r5dnzd5/1
Related research article	F. Reyes, T. DeJong, P. Franceschi, M. Tagliavini, D. Gianelle, Maximum growth potential and periods of resource limitation in apple tree, Frontiers in Plant Science 7 (2016). doi:10.3389/fpls.2016.00233

## Value of the Data

• The dataset allows analysis of the impact of fruit load, on vegetative and reproductive growth, also in respect to pruning severity, branch diameter and height in the canopy.
• The dataset can be used to infer maximum potential growth curves, periods of resource limitation for reproductive and vegetative organs.
• The dataset can be used to calibrate allometric relationships and extract several additional growth parameters.
• The structure of the dataset allows for hierarchical data analysis.
• The presented data can be used by field scientists, statisticians and modelers working on tree growth.

## 1 Objective

This paper reports a dataset obtained comparing the impact of contrasting carbon assimilate availability on the growth of different apple tree organs. Fifteen trees were divided in three groups: trees in normal field conditions (FRU), trees with low competition for carbohydrates thanks to heavy thinning (THI), trees in which the competition for carbohydrates was reduced to a minimum via the complete removal of the reproductive organs (DEF). Growth analysis of the dataset permitted the assessment of the resource limited growth periods for organs under normal field conditions. Growth data were also used to estimate maximum potential growth parameters, such as maximum potential absolute and relative growth rates [1]. This information is essential to calibrate source-sink carbon allocation models (such as in [2,3]), as well as to estimate several allometric relationships and plant growth parameters.

## 2 Data Description

**Fig. 1:**
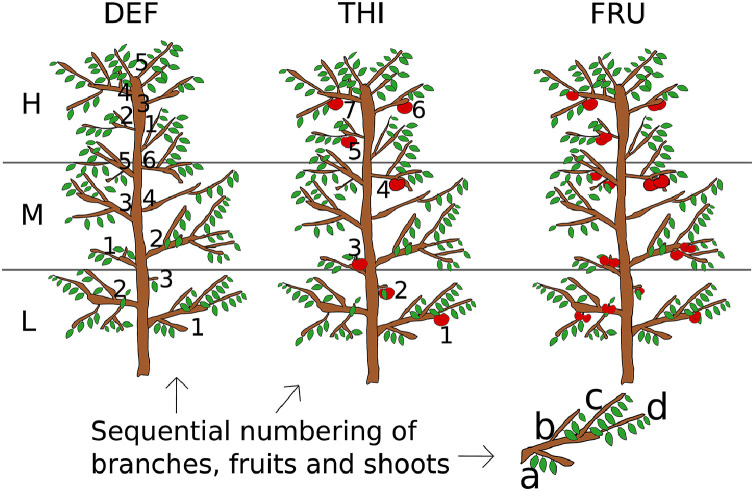
Tree treatments (DEF, complete fruit removal; THI, heavy fruit thinning; FRU, normal field conditions) and sequential numbering of tagged organs (Modified from Fig. 1 in [1]).

**Fig. 2:**
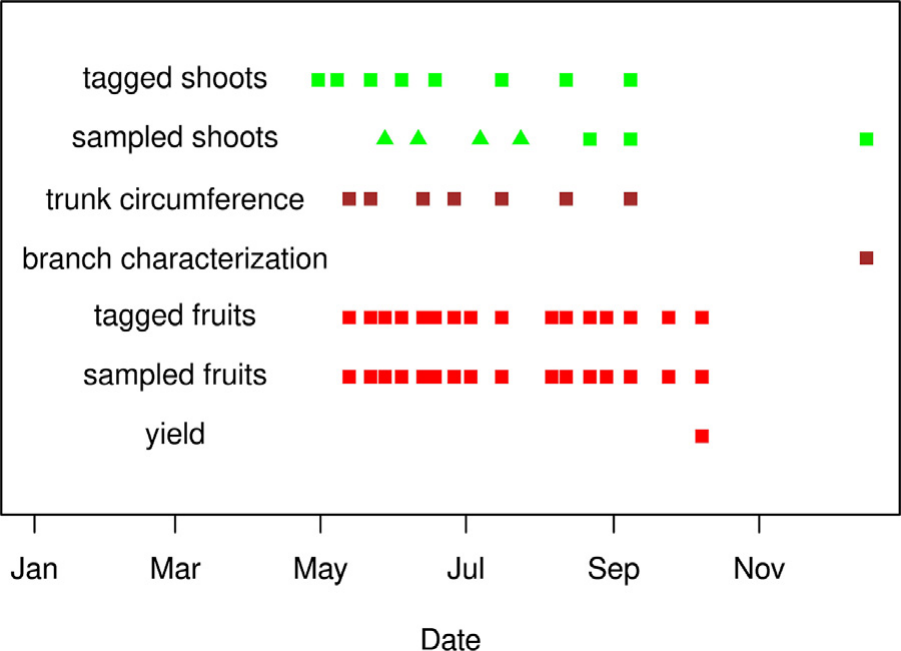
Measurement and sampling dates for the different organs from year 2014 (squares) and 2015 (triangles).

The dataset [3] is composed by an ensemble of eight spreadsheets concerning the seasonal growth of the apple tree (*Malus domestica*, Fuji Variety grafted on M9 rootstock) organs growing under three sharply different carbon assimilate availability conditions (trees in normal field conditions, FRU; in which fruit competition for carbohydrates was minimized by heavy thinning, THI; and in which vegetative competition was minimized by complete fruit removal, DEF), in the Trentino Alto Adige apple production area, Northern Italy (Fig. 1). The dataset covers both primary and secondary shoot growth, secondary trunk growth, fruit growth. It includes monitoring of tagged plant organs and a concurrent characterization of analogous sampled organs, following the same treatments (Table 1, Fig. 2). Both the monitored and sampled organs contain information useful for hierarchical data treatment, such as tree, branch and shoot numbers or canopy level (Low, Middle, High). The spreadsheets are provided in the CSV format and include treatment, sampling date, the day of the year of the measurements, and the corresponding daily accumulated growing degree days, calculated from air temperature.

**Table 1:** Variables measured in tagged and sampled organs.

Variables measured in tagged and sampled organs.				
Organ	Tagged/sampled	Numerical Variable	Total nb observations	Nb Dates
Fruit	Tagged	3 diameters	1333	16
	Sampled	3 diameters	413	16
		dry weight	413	16
Shoot	Tagged	length	4209	9
		base diameter	354	4
		number of fruits on shoot	251	1
		number of fruits on carrying branch	251	1
		height of carrying branch	449	1
		diameter of carrying branch	440	1
		pruning intensity of carrying branch	449	1
	Sampled	length	182	6
		base diameter	182	6
		apical diamater	182	6
		number of leaves	182	6
		leaf dry weight	182	6
		stem dry weight	182	6
	sampled at end of growing season	fresh mass	40	1
		volume	40	1
Trunk	Tagged	circumference	70	7

### 2.1 Spreadsheets name and content

Tagged_Fruit: contains measurements of three orthogonal diameters for nine fruits from each of five FRU and five THI trees in 16 dates.

Sampled_fruits: contains measurements of three orthogonal diameters, dry weight and canopy level for 413 fruits, sampled in 6 dates (of which 238 FRU and 175 THI; 358 from ancillary trees and 55 tagged fruits after harvest). It contains also estimates of fruit volume and density.

Fruit_yield: contains yields for five FRU and five THI trees.

Tagged_Shoot: contains measurements of length for about 20% shoots of five FRU and five DEF trees (468 shoots) in 9 dates. It also includes: a monitoring of the basal diameters for about 10 shoots per tree in four dates, shoot orientation (North/South); the number of fruits present on individual shoots and on their carrying branch as for one date in early June; the height of the carrying branch at its insertion point into the trunk, its diameter and a measure of pruning intensity evaluated after the end of the growing season.

Sampled_shoots: contains length, basal and apical diameter, number of leaves, leaf and shoot dry weight and canopy level of 182 shoots sampled from ancillary FRU trees on 6 dates.

Sampled_shoots_End_Growing_season: contains shoot fresh mass, volume and the calculated density of 40 shoots (20 FRU and 20 DEF) sampled after the end of the growing season.

Tagged_Trunk: contains measurements of trunk circumferences of five FRU and five DEF trees in 7 dates.

DegreeDays: contains the growing degree days and accumulated growing degree days from the date of full bloom, after cut off of temperatures below 5°C and below 35°C, for year 2014 in the study site.

## 3 Experimental Design, Materials and Methods

### 3.1 Field description

Field monitoring and sampling was performed in an intensive orchard located in the apple production area of the Adige river floodplain, Caldaro municipality, Trentino Alto Adige region, Italy (46° 21’ N, 11° 16’ E; 240 m a.s.l.). Trees were planted in year 2000, trained as spindelbush and spaced 1 m along the tree rows and 3 m between the rows. Orchard management followed organic guidelines, including winter pruning and tree topping at 3.6 m. A superficial water table and a drip irrigation system assured no water stress, while the availability of soil phosphorous, nitrogen and exchangeable potassium was at optimal levels [1]. Fifteen trees were selected along a tree row and randomly split in three classes: unmanipulated trees (FRU), thinned trees (THI) and defruited trees (DEF). FRU trees followed common practices for commercial orchard and hold an average of 0.47 fruits per shoot or spur. Both THI and DEF trees underwent fruit thinning on 23^rd^ April 2014 (150 GDD – Growing Degree Days after bloom). THI trees were heavily thinned, so that about 10-15 fruits were left per tree, with no more than one fruit per bourse shoot or spur. Conversely, fruits were completely removed in DEF trees. Mean FRU tree yield (26.2 kg/tree) was above the average yield at orchard level in previous years (Max = 22.3 kg/tree in years 2009-2012). A meteorological station was installed in the field, measuring mean daily air temperature at 2 m height.

### 3.2 Fruit growth

Nine fruits were tagged along a vertical transect comprising the whole tree height, on each FRU and THI tree. Three orthogonal largest diameters were measured on each tagged fruit using a digital caliper: the largest transversal diameter, a second transversal diameter obtained by turning the fruit by about 90 degrees along its longitudinal axis, and a diameter passing from the petiole to the fruit bottom, so that the fruit dimensions could be acquired similarly across different fruits and dates. Measurements were repeated about weekly. Fruits volume was estimated using the formula for a rotational spheroids (Volume = 4/3 π r1 r2 r3).

About 15 or more additional fruits were sampled from the different canopy levels on each date, alternately from additional FRU or THI ancillary trees. Finally, tagged fruits were also collected at harvest time. Sampled fruit diameters, volumes and dry weights (after petiole removal) were determined using a precision scale, after drying at 70 °C until constant weight was reached.

### 3.3 Shoot growth

For each tree, the trunk was marked at 1.20 and 2.40 m height, dividing it horizontally in three levels (Low, Medium, High). From the lower part to the top of each level, one first order branch every five was tagged and numbered sequentially.

On each tagged branch, from the insertion point in the main axis to the branch apex, every shoot and spur were tagged and lettered sequentially. The length of each tagged shoot/spur, from its insertion point in the carrying branch to its apex, was measured with a tape measure, every about two weeks from April to July and once a month in August and September in year 2014.

Secondary shoot growth was monitored on about 10 tagged shoots for each DEF and FRU trees, by measuring the basal diameters, respectively above the shoot bases on four dates (mid-July, mid-August, early-September and after the end of the growing season).

About 30 vegetative shoots were also sampled from additional FRU trees according to a stratified sampling, taking into account level, orientation and shoot length. Sampling occurred in six dates (DOY 148, 162, 188, 205, 234, 251) from years 2014 and 2015. Leaves were separated from their shoots by means of a lancet and counted. The shoot basal diameters were measured 1 cm above their bases, by means of a digital caliper. Leaves and shoots fresh weight were determined. They were then dried in a stove at 70 °C until constant weight was reached, brought to room temperature in a desiccator and weighted again. Shoots and leaves dry weights were obtained using a precision scale. 20 additional shoots per treatment were also sampled after fruit harvest. Their volume was obtained by submersion in water in a graduated cylinder, and their dry weight obtained after oven drying at 70 °C. Shoot density was calculated as the ratio between shoot dry weight and volume.

The number of fruits present on each tagged branch and on the monitored shoot/spur contained in them was also counted on 18^th^ June 2014.

The height and diameter of each tagged branch were respectively measured with a tape measure and a digital caliper by the end of the vegetative season. On the same date, the branches/shoots cross sections left exposed after the winter pruning from previous years were visually compared to the cross section of the carrying branch. Branches were then classed according to four pruning intensities: 1, 2 and 3 for cross section areas respectively smaller, equal and larger than the area of the cross section of the carrying branch; 0 for no evidences of pruning. Diameters were measured next to the insertion point into the main axis (< 1 cm far), except when pruning had occurred next to the basis of the branch. In this latter case, measurements were made just following the pruning.

The dataset presents a few missing measurements: lengths and diameters (< 1 %) when shoots/spurs were difficult to find because of occlusion due to the high leaf density; branch diameters, when they originated directly from the tree main axis, or were difficult to reach because too high in the canopy.

### 3.4 Trunk growth

Each DEF and FRU tree was equipped with a trunk collar, mounted at 10 cm above the grafting point (about 40 cm above the ground). Measurements of trunk circumference were also taken every two weeks.

## Ethics Statements

The authors declare that there are no ethical issues with the data presented, nor with the work performed to acquire them. The authors also declare that no humans or animals, nor any social media platform were subjects of this work.

## CRediT Author Statement

**Francesco Reyes:** Conceptualization, Methodology, Investigation, Data Curation, Writing. **Massimo Tagliavini:** Supervision, Funding acquisition. **Damiano Giannelle:** Resources, Supervision, Funding acquisition.

## Declaration of Competing Interest

The authors declare that they have no known competing financial interests or personal relationships that could have appeared to influence the work reported in this paper.

## Data Availability

A hierarchical dataset of vegetative and reproductive growth in apple tree organs under conventional and non-limited carbon resources (Original data) (Mendeley Data).
